# Electrospun PLA/DTAC Bicomponent Membranes for Low-Resistance and Antibacterial Air Filtration

**DOI:** 10.3390/polym17060767

**Published:** 2025-03-14

**Authors:** Xianzhong Wang, Qiumiao Yuan, Qiaonan Qian, Jingchao Wang, Chuyang Zhang, Huan Qi

**Affiliations:** 1 College of Textile and Apparel, Xinjiang University, Urumqi 830046, China; 107552204645@stu.xju.edu.cn (X.W.); yuanqiumiao2023@163.com (Q.Y.); chuyangzhang@126.com (C.Z.); 2 Institute of Smart & Ecological Textile, Quanzhou Normal University, Quanzhou 362002, China; qianqiaonan@stumail.qztc.edu.cn (Q.Q.); wangjingchao@stumail.qztc.edu.cn (J.W.); 3 College of Textile and Apparel, Quanzhou Normal University, Quanzhou 362002, China

**Keywords:** polylactic acid, electrospinning, DTAC, bimodal micro/nanofiber structure, antibacterial properties

## Abstract

Polylactic acid (PLA) fiber membranes fabricated through electrospinning exhibit significant potential for air filtration. However, their efficiency in filtering highly permeable particulate matter (PM) is limited, as these particles can carry various bacteria and toxic substances. To address this challenge, the dielectric properties of PLA are enhanced by incorporating dodecyl trimethyl ammonium chloride (DTAC), leading to the formation of a bimodal micro/nanofiber structure via conjugated electrospinning. This innovative structure effectively reduces air resistance while maintaining high filtration efficiency. The filtration performance, including filtration efficiency, pressure drop, long-term stability, and overall effectiveness, was systematically investigated. The results demonstrate that the conjugated electrospun filtration membrane achieves a filtration efficiency of 99.51% for PM0.3 and 99.97% for PM2.5. Additionally, it exhibits a high-quality factor (0.0555 Pa⁻^1^ for PM0.3 and 0.0846 Pa⁻^1^ for PM2.5), long-term stability (with PM0.3 efficiency decreasing by only 2.78% and PM2.5 efficiency decreasing by 0.01% after two months), and excellent bactericidal effects against *E. coli* and *S. aureus* due to the incorporated DTAC. Therefore, this method not only enhances filtration efficiency and reduces filtration resistance but also provides an effective approach for developing efficient filtration materials with antibacterial properties.

## 1. Introduction

Respiration is a continuous physiological process essential for maintaining normal bodily metabolism throughout life. However, the presence of particulate matter (PM) in the atmosphere, such as PM0.3 and PM2.5, has been shown to carry various bacterial contaminants that can compromise human health [1,2]. The rapid technological advancements in modern society have inevitably led to increased pollution levels, underscoring the critical need for environmental preservation. To effectively protect our respiratory systems in daily life, practical measures include the use of masks, which can capture both solid and gaseous pollutants from the air [3]. These pollutants often harbor harmful bacteria, and the overuse of antibiotics has exacerbated the issue by fostering antibiotic resistance. This situation highlights the urgent need for the development of effective antibacterial filtration materials that are both environmentally friendly and highly efficient [4,5,6,7].

In the contemporary global market, melt-blown and electrospun nonwovens are the most prevalent filtration materials. Melt-blown nonwovens typically exhibit a relatively thick fiber diameter, ranging from 1 to 5 μm, and often require post-processing electret technology to enhance filtration efficiency and meet specific requirements. This characteristic imparts electrostatic capabilities to the middle layer of commercial masks; however, the electret charges are prone to dissipation [8,9,10]. The pursuit of enhanced filtration efficiency frequently necessitates a compromise in thickness and basis weight, consequently leading to increased material consumption [11,12]. Conversely, electrospinning technology can reduce fiber diameters to below 1 μm, achieving over 99% filtration efficiency for PM0.3 through the formation of micro/nanofibers. Nevertheless, this high-performance filtration membrane possesses a dense structure, resulting in significant air resistance [13]. However, combining ultrafine fibers and nanofibers has been shown to yield a balance between high efficiency and low pressure drop [14,15]. Among petroleum-based products, such as polypropylene, due to the non-biodegradability of these materials, biodegradable starch-based PLA with the same mechanical properties, biocompatibility, and other characteristics has become crucial [16]. In accordance with the global trend toward sustainable development, the utilization of biodegradable disposable items has become an inevitable choice, rendering environmentally friendly materials such as PLA essential. Despite ongoing research on PLA nonwovens, challenges such as limited functionality persist. Notwithstanding these issues, PLA remains the primary raw material for electrospinning [17,18,19,20].

Electrospinning technology relies on a high-voltage electric field to propel a charged solution jet, creating a nanoscale fiber network [21,22]. Conjugated electrospinning employs alternating charges to direct nanofibers onto a rotating drum, thereby forming a three-dimensional scaffold [23,24]. As layers accumulate, the residual surface charge diminishes, allowing for continuous deposition and the development of more complex structures. Under specific conditions, the combined effects of high-speed ejection and airflow can lead to the convergence of two fiber groups, ultimately forming a large-scale fiber assembly or a substantial three-dimensional nanofiber sphere.

A range of complex and diverse multi-scale nano/microfiber composite filters have been developed by researchers, including beaded, mesh, and dendritic structures. The goal is to maximize contact area and fiber density while minimizing energy consumption during airflow. Results indicate that these innovative filters achieve over 99% efficiency in capturing PM0.3 particles while maintaining filtration resistance below 200 Pa, achieving a harmonious balance between effective filtration and breathability [25,26,27,28,29,30,31,32]. Wang and his research team meticulously designed and manufactured a double-layer graded structure membrane with varying mass ratios, significantly enhancing filtration efficiency and durability through a multi-level design concept [33]. Batra and his team pioneered the development of a three-dimensional nanonetwork structure based on hexadecyl trimethyl ammonium bromide (CTAB)-modified montmorillonite (MMT) clay, which has demonstrated strong inhibition against pathogenic microorganisms such as *Escherichia coli* and *Staphylococcus aureus* [34]. Although conjugated electrospinning technology is primarily used for producing high-precision nanoyarns and highly oriented membranes, it holds considerable potential for further research and development in air filtration materials [35,36]. Given its advantages, such as an exceptionally high surface area ratio and controllable porosity, conjugated electrospinning technology is poised to become the mainstream choice for manufacturing high-performance filtration materials in the future, particularly for air purification tasks in demanding environments, which are of significant importance [37,38,39].

In this study, we introduce a facile yet effective strategy for synthesizing filter membranes based on PLA with bimodal fiber diameter distributions. These membranes exhibit superior biodegradability and antimicrobial properties. The dielectric properties of PLA are significantly enhanced by the incorporation of dodecyl trimethyl ammonium chloride (DTAC), which facilitates the formation of a bimodal micro/nanofiber structure via conjugated electrospinning. We conducted a comprehensive investigation to evaluate the impact of these membranes on filtration performance, including filtration efficiency, pressure drop, long-term stability, and antibacterial efficacy. The development of biodegradable and antimicrobial PLA filters demonstrates considerable potential for applications in healthcare and high-efficiency air filtration.

## 2. Materials and Methods

### 2.1. Materials

Polylactic acid (REVODE110, 1.25 g cm^−3^ density) was procured from Zhejiang Hisun Co., Ltd. (Taizhou, China). Ethyl acetate (EA) and N, N-dimethylformamide (DMF) were supplied by Tengzhun Biotechnology Co., Ltd. (Shanghai, China). The CAS number of EA is 141-78-6, and the CAS number of DMF is 68-12-2. Dodecyl trimethyl ammonium chloride (DTAC) was obtained from Yinen Chemical Technology Co., Ltd. (Shanghai, China), and the CAS number is 112-00-5. All chemicals were used as received without further purification.

### 2.2. Preparation of Bimodal Fiber Membrane

Polylactic acid (PLA) was dissolved in a solvent mixture of N, N-dimethylformamide (DMF) and ethyl acetate (EA) at a volume ratio of 3:7. The resulting 8.0 wt% solution was continuously stirred at 70 °C using a magnetic stirrer for 24 h. Electrospinning was subsequently performed using an electrospinning instrument (SS-X3, Beijing Yongkang Co., Ltd., Beijing, China), with each spinning run utilizing a consistent volume of 1.0 mL. The solution flow rate was adjusted to control the process parameters. Membranes produced with a dual-needle head under parallel voltage were designated as DM, while conjugate membranes produced with a dual-needle head under symmetrical positive and negative voltages were designated as DCM. The distance between the needle tip and the metal collector was maintained at 12.0 cm, and the applied electric field ranged from 10.0 kV to 15.0 kV. The charged solution was collected on a metal collector wrapped with PLA spunbonded nonwoven fabric at a rate of 120 revolutions per minute. The experimental conditions were set at a temperature of 25.0 ± 3.0 °C and a relative humidity of 50.0 ± 5.0%. Finally, the membranes were dried at 25 °C for 24 h to remove residual solvents.

### 2.3. Characterization

We selected a small piece of the film and sprayed gold first; then, we put the sample in the sample chamber and then in a vacuum; then, we adjusted the observation. The scanning electron microscope (SEM) has the characteristics of large image depth of field, strong three-dimensional sense, and high resolution. SEM was employed to examine the surface characteristics and morphology of the fibers in detail, providing valuable insights into the fiber network, including fiber interactions and pore structures. The diameter and distribution pattern of PLA nanofibers were meticulously analyzed using Nano Measure 1.2 software. Given that the accuracy of thickness is directly related to the physical performance of filtration materials, an *TB 21389-2008* professional thickness tester was utilized for rigorous testing. To ensure data reliability, each sample was measured ten times, and the average value was recorded as the final thickness measurement, thereby ensuring high precision and consistency in the experimental data. The pore size distribution, a critical indicator of breathability and permeability in filtration media, was assessed using a capillary flow porometer (CFP-461-AEXL, Porous Materials Inc., Ithaca, NY, USA). This instrument, based on the capillary principle, provides comprehensive information on pore size distribution.

This is crucial for optimizing the design and manufacturing of filtration materials. The porosity is calculated using the known mass and thickness data, based on Formula (1):

(1) $$P=\;(1-\frac{Basis\;Weight}{Thickness\times \rho })\times 100\%$$

where *P* represents porosity, and the density of PLA is 1.25 g cm^−3^.

To conduct a comprehensive investigation into the actual efficiency of filtration devices in capturing PM, particularly concerning their performance against micro-pollutants, a specialized filter testing system has been meticulously designed and constructed. The purpose of this system is to simulate foggy conditions that closely resemble real-world environments. It employs a precise particle counter (AIRHUG-CP-15, Beijing Yishan Technology Co., Ltd., Beijing, China) to accurately measure PM concentrations, thereby assessing the effectiveness of the filtration medium in reducing PM concentrations on both sides of the smoke produced by cigarette combustion.

### 2.4. Assessment of Filtration Performance

The filtration efficiency and aerodynamic resistance of filtration materials were evaluated using an automatic filter tester (DR251XL, Wenzhou Darong Textile Instrument Co., Ltd., Wenzhou, China). This instrument is specifically designed to assess the performance of air filtration media, providing detailed data for analysis. During testing, a 2.0 wt% sodium chloride (NaCl) solution was utilized to generate charge-neutralized aerosol particles via a collision nebulizer. After drying, the particles were introduced into the filter test unit. The particle size distribution of the sodium chloride aerosol follows a normal distribution pattern, with a count median diameter fixed at approximately 75 nm and a geometric standard deviation maintained at 1.86, ensuring the consistency and representativeness of the test samples. Subsequently, the aerosol particles were introduced into the filter holder and passed through a 100 cm^2^ test filtration medium at a standard airflow rate of 85.0 L/min. Throughout the process, a photometer continuously monitored the NaCl aerosol concentration both upstream and downstream of the filter to compare and analyze changes in filtration efficiency. Filtration efficiency (*η*) is calculated using the following (2):

(2) $$\eta =\frac{C_{\mathrm{up}}-C_{\mathrm{down}}}{C_{\mathrm{up}}}\times 100\%$$

where C_up_ and C_down_ represent the concentration of NaCl aerosol upstream and downstream, respectively.

The quality factor (QF) is considered to be a comprehensive parameter of filtration efficiency and pressure drop. The quality factor was calculated using Formulas (3) and (4).

(3) $$\Delta P=P_{1}-P_{2}$$

(4) $$\mathrm{QF}=-\frac{\mathrm{Ln}\left(1-\eta \right)}{\Delta P}$$

where ΔP represents the pressure drop.

The filtration performance of the materials was evaluated across five distinct regions through testing, and an average value was subsequently used as the final data for assessment. Additionally, the filtration efficiency of each material was re-evaluated after a two-month storage period to assess its stability over time.

### 2.5. Evaluation of Antibacterial Performance

The antibacterial activity of the filtration membrane composed of PLA and DTAC was evaluated in accordance with the provisions of *TB 15979-2002*. The assessment employed the shaking method to determine the sample’s antibacterial efficacy against *Escherichia coli* (*E. coli*, ATCC 25922, a Gram-negative bacterium) and *Staphylococcus aureus* (*S. aureus*, ATCC 25923, a Gram-positive bacterium). The antibacterial rate was calculated using the colony counting method, and the antibacterial rate was calculated using Formula (5):

(5) $$R=\frac{A_{0}-A_{1}}{A_{0}}$$

where A_0_ represents the number of colonies in the control group, and A_1_ represents the number of colonies in the test group.

## 3. Results and Discussion

### 3.1. Design of Bimodal PLA Structure

**Figure 1:**
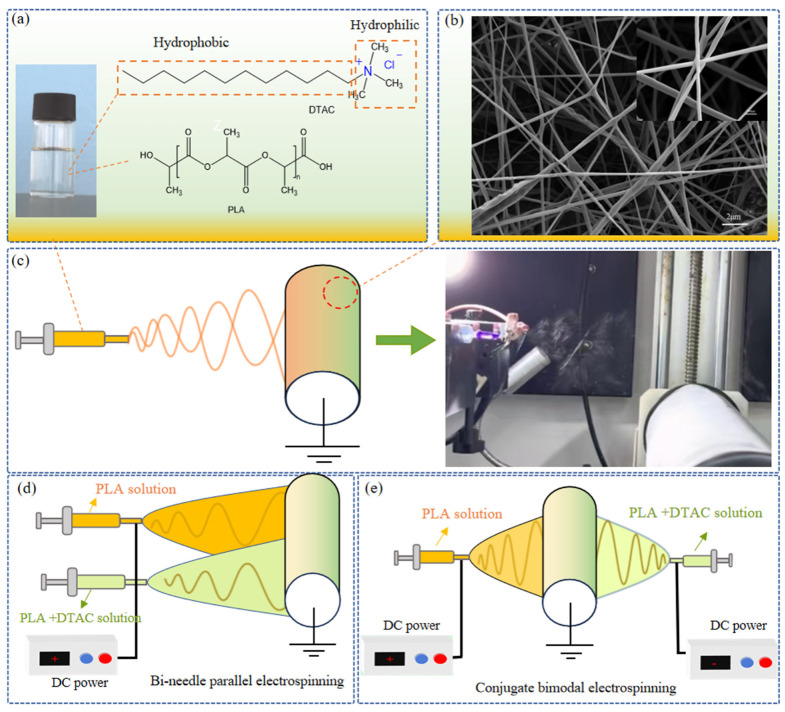
(**a**) Spinning solution composition. (**b**) Morphology of the spinning fibers. (**c**) Schematic diagram of electrospinning device. (**d**) Bi-needle parallel electrospinning. (**e**) Conjugate bimodal electrospinning.

Currently, the primary filtration materials utilized are melt-blown and electrospun membranes. To achieve high protection, low resistance, and high filtration efficiency with long-term stability, we have developed a biodegradable PLA filtration material using environmentally friendly organic solvents to minimize environmental impact. The bimodal antibiofilm structure is illustrated in Figure 1. The design of the film adheres to the following principles: (1) PLA, a polymer derived from biomass feedstocks such as corn starch, exhibits excellent biocompatibility and degradability. The use of organic solvents, as opposed to traditional industrial chemical solvents, significantly reduces the risk of environmental contamination. (2) A bactericidal filtration layer has been developed to effectively inhibit the survival and spread of pathogens, thereby ensuring the safety and health of users. (3) By employing a two-nozzle conjugate electrospinning system, we achieve precise control over the fiber network, creating an efficient filtration barrier while maintaining low resistance and excellent air permeability. To meet these requirements, we prepared a bimodal filter with antibacterial activity, consisting of submicron fibers (191 ± 21 nm) and nanofibers (76 ± 16 nm). This composite structure ensures high filtration efficiency, low pressure drop, and effective antibacterial properties.

### 3.2. Morphological Observation of Membranes

**Figure 2:**
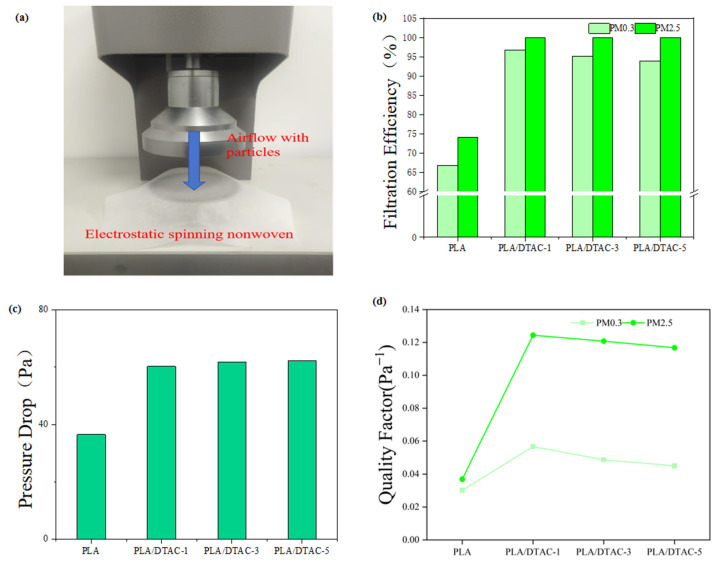
(**a**) Diagram of air filtration equipment used to test the filtration efficiency, (**b**) filtration efficiency, (**c**) pressure drop, and (**d**) quality factor of membranes with different proportions of DTAC for PM0.3 and PM2.5.

The integration of micro- and nanofiber structures has been shown to significantly enhance the air filtration performance of filtration membranes. The incorporation of DTAC improves the dielectric properties of the fibers. By electrospinning an 8.0% PLA solution doped with DTAC, the resulting fibers exhibit finer diameters, and the resultant film significantly enhances the electrostatic adsorption. As illustrated in Figure 2a, filtration efficiency was evaluated by measuring the concentration of sodium chloride aerosol particles both upstream and downstream of the membrane. To ensure consistent properties of the final electrospun film, a fixed volume of solvent (0.5 mL) was utilized during performance testing. Figure 2b–d demonstrate that, as the DTAC content increases from 1.0% to 3.0% and then to 5.0% of the solvent, the filtration efficiency, pressure drop, and QF initially rise and subsequently decline. Notably, the PLA/DTAC-1 sample exhibits optimal filtration performance, achieving a QF of 0.0566 Pa⁻^1^ for PM0.3 and an average nanofiber diameter of 111 nm, which is significantly finer than fibers produced by solution or melt electrospinning or blow spinning techniques. The increased surface area of the nanofiber structure enhances the likelihood of contact with airborne pollutant particles. The addition of DTAC plays a crucial role in improving electrostatic adsorption, facilitating more effective capture of small particles.

### 3.3. Morphology and Structural Analysis

**Figure 3:**
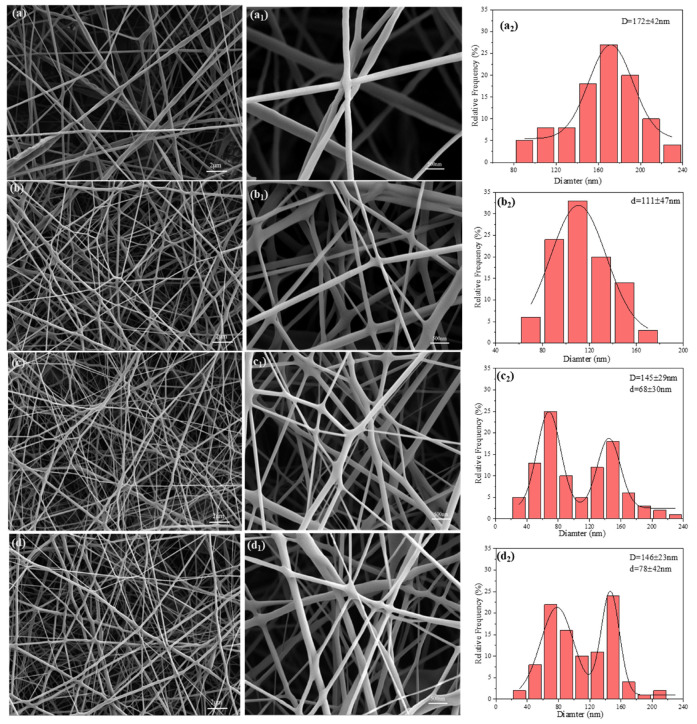
(**a**,**a_1_**,**a_2_**) PLA, (**b**,**b_1_**,**b_2_**) PLA/DTAC-1, (**c**,**c_1_**,**c_2_**) DM-PLA/DTAC-1, and (**d**,**d_1_**,**d_2_**) DCM-PLA/DTAC-1 SEM images and fiber diameter distribution graphs.

Scanning electron microscopy (SEM) was employed for a comprehensive microstructural analysis of the filtration materials. As illustrated in Figure 3a, the PLA-based fiber membrane exhibits a smooth and continuous surface, devoid of noticeable defects such as beading [40]. In its pure form, without any additives, the average fiber diameter measures approximately 172 nm, demonstrating excellent uniformity. Figure 3b indicates that the incorporation of DTAC as an additive significantly reduces the fiber diameter to 111 nm, indicating that DTAC can effectively modulate the fiber formation process, resulting in finer fibers. Despite this reduction, the fibers maintain a uniform cylindrical shape, underscoring their good controllability.

Figure 3c,d illustrate the fiber morphology and diameter distribution of DM-PLA/DTAC-1 and DCM-PLA/DTAC-1 under two different conditions. Both sets of experiments reveal a distinct bimodal structure composed of submicron fibers (146 nm) and nanofibers (78 nm). This indicates that the fiber membrane consists of fibers of varying sizes while maintaining a uniform distribution, thereby forming a complex hierarchical structure within the composite material [41]. The fiber diameter distribution curve clearly reflects the dual peak characteristics of DM-PLA/DTAC-1 and DCM-PLA/DTAC-1. Additionally, the interwoven fibers result in DCM-PLA/DTAC-1 exhibiting a more intricate three-dimensional structure, resembling fluffy cotton. By adjusting the velocity difference between the dual nozzles, the formation of fibers can be precisely controlled, allowing for the production of a highly uniform composite fiber network with a bimodal distribution.

**Figure 4:**
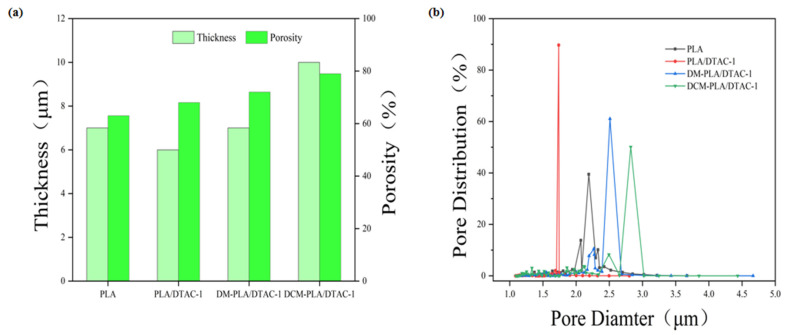
(**a**) Porosity and thickness; (**b**) pore size distribution of PLA, PLA/DTAC-1, DM-PLA/DTAC-1, and DCM-PLA/DTAC-1.

The microstructure of nonwovens is crucial for their filtration performance, as the arrangement of fibers and the resulting pore characteristics significantly influence their effectiveness. The incorporation of DTAC notably reduces the pore size of PLA electrospun membranes due to its enhanced dielectric properties, which facilitate the formation of finer fibers [15]. As illustrated in Figure 4a, a comparison between DCM-PLA/DTAC-1 and DM-PLA/DTAC-1 reveals that DCM-PLA/DTAC-1 exhibits a higher porosity of 79.51%, primarily due to its more voluminous three-dimensional structure. This difference is also evident in the thickness comparison: under identical material input conditions, the thickness of DCM-PLA/DTAC-1 is significantly greater. As illustrated in Figure 4b, the analysis of the pore size distribution indicates that these electrospun membranes exhibit a pore size range within a specific interval, typically conforming to a standard normal distribution. The DCM-PLA/DTAC-1 sample demonstrates the largest average pore size at 2.48 µm, closely followed by DM-PLA/DTAC-1 at 2.37 µm. This observation suggests that the bimodal diameter distribution of the fibers promotes the formation of larger pores, likely due to the stacking of fibers with varying thicknesses, which creates additional spatial effects. Notably, the gaps between thicker fibers are wider than those between thinner fibers, thereby facilitating the development of larger pores. For fibers exhibiting a bimodal diameter distribution, the stacking of multi-layer fibers creates a filtration medium with varying pore sizes, significantly enhancing overall filtration performance. Furthermore, the larger average pore size can improve gas flow capacity, thereby reducing pressure drop during filtration and increasing overall system efficiency.

### 3.4. Effect of Bimodal Diameter Difference on Filtration

**Figure 5:**
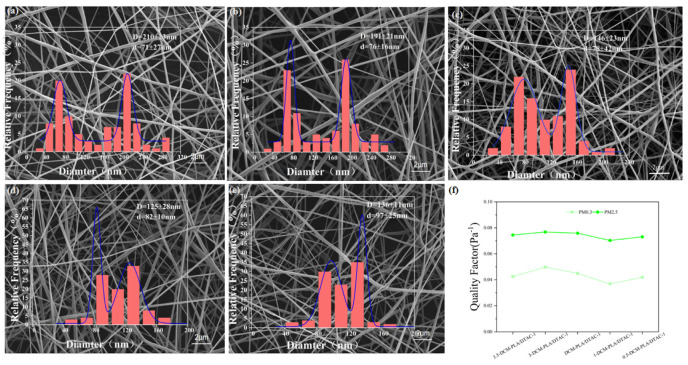
SEM images and nanofiber diameter distributions of (**a**) 3.5-DCM-PLA/DTAC-1, (**b**) 3-DCM-PLA/DTAC-1, (**c**) DCM-PLA/DTAC-1, (**d**) 1-DCM-PLA/DTAC-1, and (**e**) 0.5-DCM-PLA/DTAC-1. (**f**) The QF value of different films.

To understand the microstructural changes in conjugated electrospun membranes, scanning electron microscopy (SEM) was employed for detailed observation. The SEM images presented in Figure 5a–e demonstrate that all electrospun membranes are predominantly composed of randomly arranged fibers that range from nanoscale to microscale dimensions. These fibers interweave to form a highly interconnected and permeable porous network [38,42]. This unique structure not only increases the surface area of the membrane but also enhances its permeability to airflow and improves the capture efficiency of particulate matter. When a conductive additive, such as DTAC, is incorporated into the PLA mixture, the electrical conductivity of the membrane significantly increases, which directly affects fiber refinement. Adjusting the speed differential between the spraying and collecting devices, particularly the advancing speed, is a critical factor in regulating fiber diameter, as summarized in Table 1. To ensure consistent properties of the final electrospun film, a fixed volume of solvent (0.5 mL) was utilized during the film spinning process for performance testing. Figure 5f illustrates that, under identical conditions, an optimal filtration effect is achieved with bimodal fiber diameters of 191 nm and 76 nm, resulting in a QF value of 0.0498 Pa^−1^ for PM0.3 and 0.0768 Pa^−1^ for PM2.5. As the bimodal difference increases further, the filtration effect does not improve linearly; instead, it enters a complex phase of change. Initially, a moderate increase in bimodal difference enhances the membrane’s filtration efficiency, as the diversification of the porous structure increases the opportunities for particulates to contact the fibers, thereby improving capture efficiency. However, if the bimodal difference becomes excessively large, it may lead to an overly loose fiber network. Conversely, reducing the bimodal difference results in more uniform fiber diameters, approaching a nearly monodisperse distribution state [43]. The increase in DTAC content also enhances filtration performance by modifying the static charge distribution among the fibers, improving fiber adhesion, and increasing the overall stability of the membrane. Furthermore, thinner fiber layers can enhance the interception of small particles, allowing the membrane to maintain high permeability while demonstrating superior filtration capabilities. At the same time, the filtration efficiency of single and double peaks is influenced by additional factors such as fiber junction formation and fiber density. The formation of junctions between fibers reduces the pore size between them. This change directly enhances the filter material’s ability to intercept smaller particles, thereby improving filtration accuracy. However, these junctions can also impede fluid flow through the filter material, leading to increased resistance. This increase in resistance is attributed to the altered pore structure between fibers, which complicates and tortures the fluid flow path. Fiber density significantly impacts filtration efficiency. Higher fiber density implies a greater number of fibers per unit area, consequently reducing the inter-fiber gaps. Smaller gaps enhance the capture and interception of small particles and impurities, thus improving filtration accuracy. Nonetheless, while increased fiber density improves filtration accuracy, it also introduces higher filtration resistance due to the increased obstruction of fluid flow by more fibers. The fiber density is particularly obvious in the single peak.

### 3.5. Filtration Performance and Mechanism

**Figure 6:**
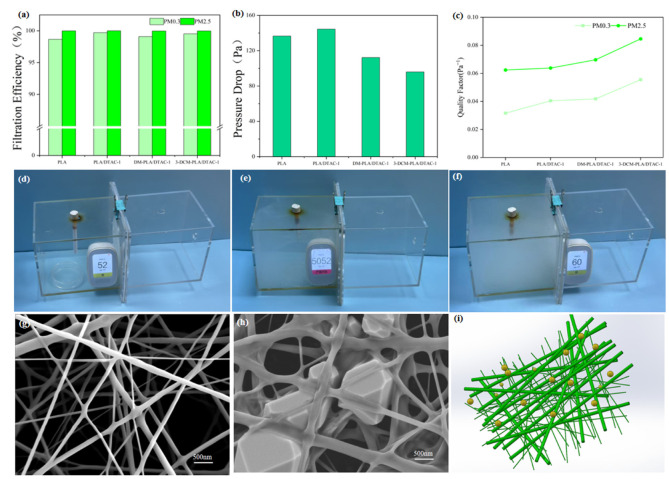
(**a**) Filtration efficiency, (**b**) pressure drop, and (**c**) quality factor of PLA, PLA/DTAC-1, DM-PLA/DTAC-1, and 3-DCM-PLA/DTAC-1. (**d**) Filter test system for burnt cigarette particles; (**e**,**f**) determination of the amount of PM blocked by 3-DCM-PLA/DTAC-1 filter membrane; (**g**,**h**) filter before and after to block smoke; (**i**) conjugate filtration mechanism diagram.

Electrospun nonwovens are well known for their exceptional capacity to capture particles, particularly ultra-fine particles such as PM0.3, demonstrating remarkably high filtration efficiencies. This study concentrates on the development of filtration membranes that not only sustain high filtration efficiency but also significantly decrease air resistance, a critical factor in the advancement of air filtration technology. The experimental results, as illustrated in Figure 6a,b, demonstrate that, at a constant airflow rate of 85.0 L/min, various types of membranes exhibited remarkable performance in filtering PM0.3 particles. Specifically, the filtration efficiencies for PLA, PLA/DTAC-1, DM-PLA/DTAC-1, and 3-DCM-PLA/DTAC-1 were recorded at 98.66%, 99.71%, 99.08%, and 99.51%, respectively. Correspondingly, the pressure drops for these four membrane samples were measured at 136.5 Pa, 144.4 Pa, 112.2 Pa, and 95.9 Pa. Notably, the membrane with a bimodal fiber diameter distribution demonstrated particularly exceptional performance, with the pressure drop reduced to 112.2 Pa. The application of conjugated bimodal spinning technology further decreased the resistance to 95.9 Pa, underscoring the potential of this technology in minimizing filtration resistance. As illustrated in Figure 6c, the graphical data indicate that the fluffy and uneven fiber structure contributes to a QF value of 0.055 Pa^−1^. This enhancement is attributed to the effective interception by the nanofibers, which significantly improves filtration efficiency. By creating a three-dimensional structure with a loose texture, conjugated electrospinning technology once again demonstrates its superiority in facilitating airflow [44]. The strategic combination of fiber diameter and fluffiness ultimately results in a substantial reduction in pressure drop.

In addition to smoke removal efficiency, the ability to eliminate particulate matter larger than PM2.5 is a critical indicator for evaluating the performance of filtration materials. To assess the effectiveness of the 3-DCM-PLA/DTAC-1 membrane in practical applications, a simulation test was specifically designed. In this test, a cigarette was ignited at one end, and the 3-DCM-PLA/DTAC-1 membrane was positioned between two transparent chambers. Ventilation circulation was then initiated, allowing for real-time observations of smoke accumulation on both sides of the membrane. As the cigarette burned, smoke gradually filled the left chamber, and PM2.5 counters were deployed on both sides of the membrane to accurately measure particulate concentrations. As illustrated in Figure 6d–f, it is evident that 3-DCM-PLA/DTAC-1 effectively intercepts and adsorbs PM, resulting in a remarkably clear and transparent right chamber. The PM2.5 concentration recorded in the left chamber reached 5052 μg/m^3^. Figure 6g,h demonstrate that 3-DCM-PLA/DTAC-1 not only efficiently captures large suspended particles in the air but also addresses common challenges associated with smoke purification in everyday life, thereby providing robust respiratory safety, particularly for particulate matter exceeding the PM2.5 threshold. The fluffy bimodal distribution characteristic creates a three-dimensional network architecture with an interlaced structure, as illustrated in Figure 6i. This distinctive geometric configuration endows the material with extensive spatial dimensions, significantly increasing the airflow path. Consequently, there are more opportunities for particles to interact with the fiber surface, thereby enhancing capture efficiency [45,46,47]. Specifically, as air navigates the complex pathways of the fiber network, the likelihood of collisions between particles and fibers increases, which accelerates the sedimentation and retention processes, ensuring effective filtration.

### 3.6. Load Performance and Durability

**Figure 7:**
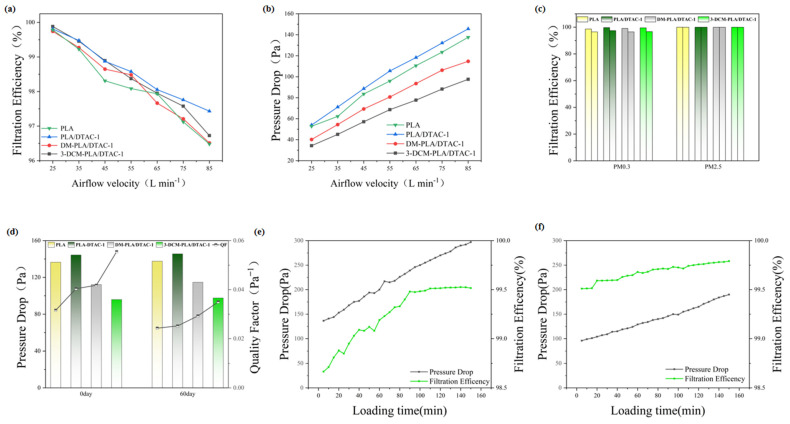
(**a**) Filtration efficiency at various airflow rates; (**b**) pressure drop of PLA, PLA/DTAC-1, DM-PLA/DTAC-1, and 3-DCM-PLA/DTAC-1 at different airflow rates; (**c**) filtration efficiency for PM0.3 and PM2.5 after 60 days of storage; (**d**) pressure drop and quality factor for PM0.3 and PM2.5 after 60 days of storage; (**e**) filtration efficiency and pressure drop of pure PLA during the loading test; (**f**) filtration efficiency and pressure drop of 3-DCM-PLA/DTAC-1 during the loading test.

In the long-term evaluation of PLA membrane filtration performance, there was a slight decrease in filtration efficiency; however, this change still significantly exceeded the standard requirements. As illustrated in Figure 7a,b, after two months of storage, when the airflow rate was reduced from 85 L/min to 25 L/min, the PM0.3 filtration efficiency of the membrane increased from 96.73% to 99.89%. This indicates that the PLA membrane can sustain high filtration efficiencies across varying flow rates, with particularly notable improvements at lower flow rates. Furthermore, a positive correlation exists between flow rate and pressure drop: as the flow rate decreases, the pressure drop also diminishes. The pressure drop decreased from 97.5 Pa to 34.3 Pa, demonstrating that the PLA membrane can maintain low resistance even after prolonged use, thereby enhancing air circulation efficiency.

As illustrated in Figure 7c, after two months, all membranes exhibited a slight decrease in PM0.3 filtration efficiency. This decline can be attributed to the gradual dissipation of surface charge on the membrane over time, which adversely affects filtration performance. In contrast, the PM2.5 filtration efficiency showed minimal change and remained consistently high. This observation suggests that the filtration of PM2.5 particles primarily relies on mechanical interception rather than electrostatic adsorption. Figure 7d illustrates that the QF value decreased from 0.056 to 0.035 Pa^−1^, indicating that the filtration material continues to exhibit strong filtration performance despite long-term storage. This finding underscores the exceptional storage stability of the filtration material, with the filtration efficiency of 3-DCM-PLA/DTAC-1 remaining at 96.73%. Figure 7e,f present a comprehensive comparison of the performance differences between the pure PLA film and the modified 3-DCM-PLA/DTAC-1 film in the loading experiment, with a particular emphasis on dust holding capacity and durability. The dust holding capacity of the 3-DCM-PLA/DTAC-1 film reaches 8.93 g/m^2^, surpassing that of other electrospun films. This enhanced capacity can be attributed to the three-dimensional structure of the conjugated film, which facilitates the capture of particles in the deeper layers, rather than relying solely on surface capture.

The 3-DCM-PLA/DTAC-1 film exhibits a lower rate of resistivity growth and takes longer to double its initial resistivity, indicating an extended service life. This film not only enhances dust-holding capacity but also slows the rate of resistivity increase, thereby improving its suitability for long-term use. Overall, due to the larger volume and weight of particulate matter, interception and gravitational effects play a more significant role in capturing these particles. Consequently, even after prolonged use and storage, the filter media can maintain effective filtration performance, primarily due to the predominant influence of mechanical interception effects.

### 3.7. Antimicrobial Performance

**Figure 8:**
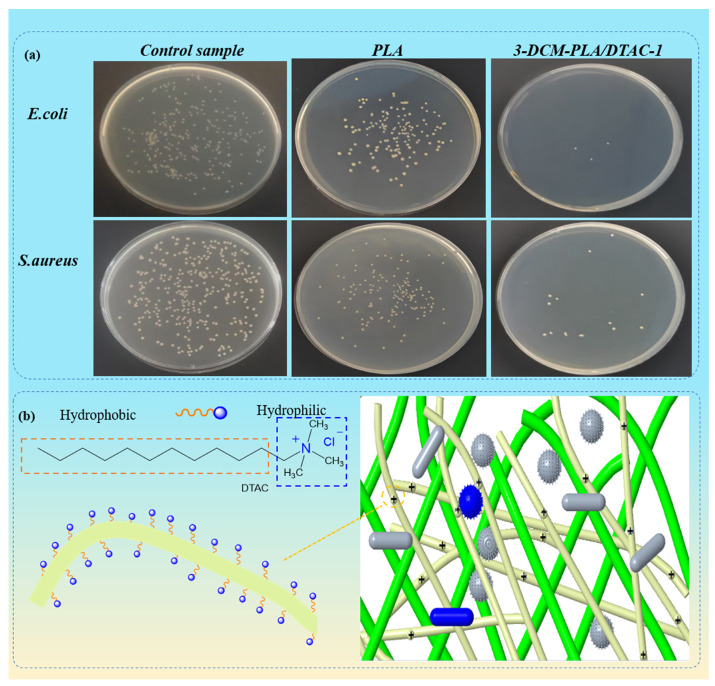
(**a**) The antibacterial properties of PLA and 3-DCM-PLA/DTAC-1 against *Escherichia coli* (*E. coli*) and *Staphylococcus aureus* (*S. aureus*). (**b**) Antibacterial mechanism of conjugated electrostatic spinning containing DTAC.

In this study, we evaluated the antimicrobial efficacy of samples by quantitatively assessing the antimicrobial activity of PLA-based materials containing DTAC against Gram-negative *Escherichia coli* (*E. coli*) and Gram-positive *Staphylococcus aureus* (*S. aureus*) bacteria. As illustrated in Figure 8a, PLA filter membranes exhibit some antimicrobial properties against both *E. coli* and *S. aureus*; however, the antimicrobial performance of 3-DCM-PLA/DTAC-1 is significantly superior to that of pure PLA. By calculating the antimicrobial rate using the enumeration method, the inhibition rates of PLA against *E. coli* and *S. aureus* are 53.66% and 46.44%, respectively, while those of 3-DCM-PLA/DTAC-1 are 99.05% and 95.35%, respectively.

As illustrated in Figure 8b, the structure of DTAC and the killing mechanism of the bimodal filter on bacteria are demonstrated. Here, the antibacterial mechanism of DTAC and its effectiveness in a double-layer filtration system are introduced in detail. DTAC has hydrophilic and hydrophobic groups, while the hydrophilic group has a positive charge on one side, which interacts electrostatically with negatively charged bacteria. The positive charge of DTAC molecules attracts bacteria to the filter, facilitating its removal [48]. On the other side is the hydrophobic alkyl group, which can also interact with the hydrophilic group of bacterial cells, affect the permeability of the membrane, cause lysis, destroy the cell structure, and cause the dissolution and death of the cell.

## 4. Conclusions

In conclusion, by implementing a conjugated electrospinning process, we successfully prepared nanofiber membranes with a bimodal diameter distribution. This membrane leverages the exceptional properties of PLA, the enhanced dielectric performance of DTAC, and the unique structure of coexisting coarse and fine fibers, resulting in remarkable filtration efficiency, low airflow resistance, and antibacterial activity. The variation in fiber diameters, along with the corresponding changes in specific surface area, collectively influences the filtration performance of the fiber membrane, leading to significant improvements across all metrics. At the structural design level, the bimodal fiber filter comprises continuous nanofibers (average diameter 76 ± 16 nm) and sub-micron fibers (average diameter greater than 191 ± 21 nm). Even at such a fine scale, this material maintains a filtration efficiency exceeding 99.51%, with a pressure drop of only 95.9 Pa and a QF of 0.0555 Pa^−1^. Importantly, the bacterial filtration efficiency exceeded 95%, underscoring its significant potential in biological safety applications. Notably, even after two months of storage, the filtration performance remained high, exhibiting only minor degradation. These research findings have substantial implications for the development of new nanofiber materials.

## Figures and Tables

**Table 1:** Preparation of fiber membranes with different advancing speeds in conjugation mode.

Sample	Positive PLA Liquid Supply Rate (mL/h)	Positive PLA/DTAC Liquid Supply Rate (mL/h)
3.5-DCM-PLA/DTAC-1	3.5	0.5
3-DCM-PLA/DTAC-1	3	1
DCM-PLA/DTAC-1	2	2
1-DCM-PLA/DTAC-1	1	3
0.5-DCM-PLA/DTAC-1	0.5	3.5

## Data Availability

The data presented in this study are available on request from the corresponding author (due to privacy).
